# Tumor-Associated Macrophages and Neutrophils in Tumor Microenvironment

**DOI:** 10.1155/2016/6058147

**Published:** 2016-02-04

**Authors:** Jaehong Kim, Jong-Sup Bae

**Affiliations:** ^1^Department of Biochemistry, School of Medicine, Gachon University, Incheon 406-799, Republic of Korea; ^2^College of Pharmacy, CMRI, Research Institute of Pharmaceutical Sciences, BK21 Plus KNU Multi-Omics based Creative Drug Research Team, Kyungpook National University, Daegu 702-701, Republic of Korea

## Abstract

Distinct tumor microenvironment forms in each progression step of cancer and has diverse capacities to induce both adverse and beneficial consequences for tumorigenesis. It is now known that immune cells can be activated to favor tumor growth and progression, most probably influenced by the tumor microenvironment. Tumor-associated macrophages and tumor-associated neutrophils can exert protumoral functions, enhancing tumor cell invasion and metastasis, angiogenesis, and extracellular matrix remodeling, while inhibiting the antitumoral immune surveillance. Considering that neutrophils in inflammatory environments recruit macrophages and that recruited macrophages affect neutrophil functions, there may be various degrees of interaction between tumor-associated macrophages and tumor-associated neutrophils. Platelets also play an important role in the recruitment and regulation of monocytic and granulocytic cells in the tumor tissues, suggesting that platelet function may be essential for generation of tumor-associated macrophages and tumor-associated neutrophils. In this review, we will explore the biology of tumor-associated macrophages and tumor-associated neutrophils and their possible interactions in the tumor microenvironment. Special attention will be given to the recruitment and activation of these tumor-associated cells and to the roles they play in maintenance of the tumor microenvironment and progression of tumors.

## 1. Introduction

**Figure 1:**
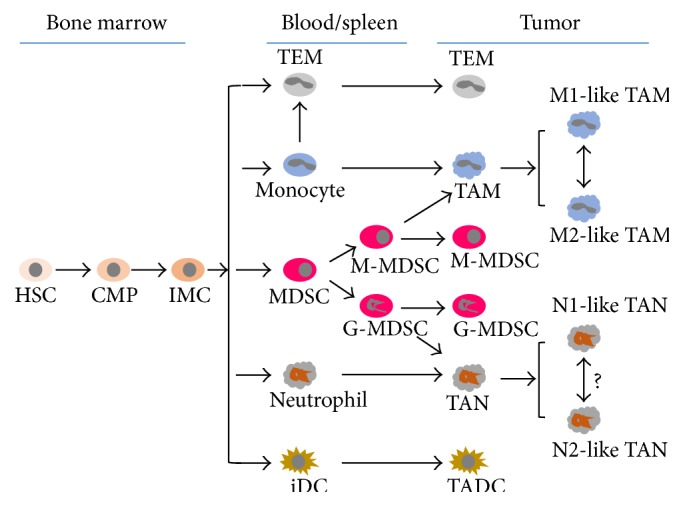
Differentiation of tumor-associated myeloid cells begins from hematopoietic stem cells (HSC) in the bone marrow. CMP: common myeloid progenitors, IMC: immature myeloid cells, TEM: Tie2-expressing monocyte, MDSC: myeloid-derived suppressor cell, M-MDSC: myeloid MDSC, G-MDSC: granulocytic MDSC, iDC: immature dendritic cells, TADC: tumor-associated dendritic cells, TAM: tumor-associated macrophage, and TAN: tumor-associated neutrophil [63].

Cancer-related nonresolving inflammation in the tumor microenvironment (TME) is a hallmark of cancer, and cancer cells are confronted with various types of stromal and immune cells across all stages of the disease, from early carcinogenesis to tumor progression and metastasis [1, 2]. The progression of cancer has traditionally been regarded as a multistep process with genetic and epigenetic changes targeting only cancer cells. However, studies over the past two decades have revealed that the TME is an equally important determinant of tumor behavior. The components of the TME include local stromal cells, such as resident fibroblasts and macrophages, and distant recruited cells such as endothelial cells, immune cells including myeloid and lymphoid cells, bone marrow-derived precursor cells, and circulating platelets. To note, tumor-associated myeloid cells (TAMCs) comprise five distinct myeloid populations: tumor-associated macrophages (TAMs), monocytes expressing the angiopoietin-2 receptor Tie2 (Tie2-expressing monocytes or TEMs), myeloid-derived suppressor cells (MDSCs), tumor-associated neutrophils (TANs), and tumor-associated dendritic cells (Figure 1) [3]. Of these, TAMCs result in TAMs and TANs to be discussed in this review.

## 2. General Characteristics of TAMs

Macrophages are the most well-characterized type of tumor-infiltrating immune cell, and it is not surprising that they play a prominent active role from early carcinogenesis to tumor progression including metastasis [4]. While macrophages involved in cancer-initiating conditions are immune activated (e.g., antitumoral), once tumors are established, the macrophages are educated to become protumoral [5]. Currently, the majority of evidence supports a tumor-promoting role of a specific subpopulation of macrophages, TAMs within the primary TME. Surprisingly, macrophages can constitute up to 50% of a tumor mass, forming a major component of immune cell infiltrate in the TME [4, 6, 7]. This was long considered to be an indication of antitumor immunity, considering the inherent phagocytic and cytotoxic properties of macrophages. However, high frequencies of TAMs are generally associated with poor prognosis in most human cancers [8, 9], and this is in stark contrast with the traditional notion that macrophages play host-protecting roles in inflammatory microenvironments. When exposed to signals from the TME, macrophages show a surprising degree of plasticity in functional reprogramming and adopt either pro- or anti-inflammatory phenotypes in response to environmental stimuli [10]. Importantly, another tumor-promoting structure—the TME for metastasis, consisting of macrophages, endothelial cells, and tumor cells—is recognizable in metastatic sites and has been shown to be predictive of metastatic potential in human breast cancers [11]. This observation is explained by the role of TAMs in cancer cell survival through immunosuppression, invasion, metastasis, and angiogenesis. In the transition from benign to malignant invasive cancer, the TME is flooded with cytokines and growth factors. TAMs display delayed and defective NF-*κ*B activation in response to signals such as LPS and TNF-*α* and this enables TAMs to sustain “smouldering inflammation” in the TME, which is responsible for the protumor phenotypes [12].

Available information suggests that TAMs infiltrating established tumors acquire the properties of M2-like phagocytic population and phenotypes such as promotion of tumor growth and angiogenesis, remodeling of tissues, and suppression of antitumor immunity [12]. Analogously to the T helper (Th1) and Th2 dichotomy, macrophages have been classified into specific M1-like (activated) or M2-like (alternatively activated) functional status based on functional polarization by the microenvironment [13, 14]. It has been widely accepted that IFN-*γ* alone or with microbial LPS or cytokines such as TNF and GM-CSF induces classically activated M1 macrophages and immune complexes, IL-4, IL-6, IL-10, IL-13, IL-21, IL-33, and Notch can elicit the M2 form of macrophage activation [15, 16]. However, M1- and M2-polarized macrophages are extremes in a continuum in a wide range of functional states and truly polarized macrophages are rare [17, 18]. Instead, TAM can be described as M(IL-4), M(Ig), M(IL-10), M(GC: glucocorticoid), M(IFN-*γ*), M(LPS), and so forth, according to recently attempted nomenclature linked to the activation standard [19]. In turn, TAMs contribute to high IL-10 and TGF-*β* levels in the TME [20] and they express inflammatory cytokines (e.g., IL-1*β*, IL-6, IL-12, and TNF-*α*), albeit at low levels [21]. In response to stimuli from TEMs, TAMs can promote tumor growth through the production of activation factors for stromal and cancer cells (EGF, bFGF, VEGF, PDGF, and TGF-*β*) [22–25]. These findings indicate mutual interactions between TAMs and the TME for tumor progression.

Recently emerging efforts to establish a common language for describing the properties of the macrophages under investigation prefer the term “activation” rather than “polarization” for the classification of functional status of TAMs [19]. Because TAMs are not truly polarized population of macrophages, we will use the term “activation” instead of “polarization” in this review to avoid further confusions.

As macrophages in human cancer can neither be uniformly classified into classically activated M1-like or alternatively activated M2-like macrophages, they are collectively termed TAMs and the former view of TAMs as a skewed M2-like single macrophage population is an oversimplification [26]. Rather, M1- and M2-polarized macrophages are two extremes in a continuum in a wide range of functional states [17, 18, 27] and recent study with highly standardized stimulation of human macrophages showed that current M1 versus M2 polarization model can be extended to a “spectrum model” with at least nine distinct macrophage activation programs [27]. It has become clear that dynamic alterations in the phenotypes of macrophages occur during tumor initiation, progression, and metastasis and that subpopulations of TAMs are responsible for distinct tumor-promoting activities [5, 28, 29]. Notably, tumors have a diverse spectrum of disorders and the distribution and function of TAMs differ considerably in different microregions of the neoplastic tissue; recent large-scale transcriptome analyses revealed that macrophages have a mixed phenotype expressing both M1-like and M2-like markers [5, 13]. Different signals from particular locations in the TME seem to influence activation of TAMs and overall tumor prognosis [30]. For example, within cancerous tissue, TAMs can be microanatomically diverse, including the accumulation of cells with protumor properties in hypoxic areas [31] and differences in inflammatory components and pathways between tumors originating in distinct anatomical sites [31, 32]. TAMs have proangiogenic activity, and macrophage infiltration in tumors is generally associated with high vascular density [33]. M2-like TAMs, highly localized in hypoxic tumor areas, have displayed superior proangiogenic activity* in vivo*, and the numbers increased as the tumors progressed [31]. TAMs express various molecules modulating angiogenesis, such as VEGF, bFGF, TNF-*α*, IL-1*β*, CXCL8, cyclooxygenase 2, plasminogen activator (uPA), PDGF-*β*, MMP7, MMP9, and MMP12 [34]. Of note, the composition of the immune microenvironment and the overall activation state of TAMs become more favorable for tumor growth during tumor progression, and the functional roles of macrophages during tumor initiation become changed during tumor progression.

Reversion of M2-like macrophages to M1-like cells and reduction of immunosuppressive effects from the M2 population have been reported when TAMs recovered an M1 phenotype following IFN-*γ* treatment [35, 36]. These results indicate that activation of TAMs can be reversible and suggest new possible therapeutic strategies targeting reeducation of TAMs. The identification of genetic and epigenetic mechanisms [37–39] underlying macrophage diversity in tissues and their different forms of activation may pave the way to reeducation strategies.

## 3. Origin and Recruitment of TAMs in Tumor Sites

**Figure 2:**
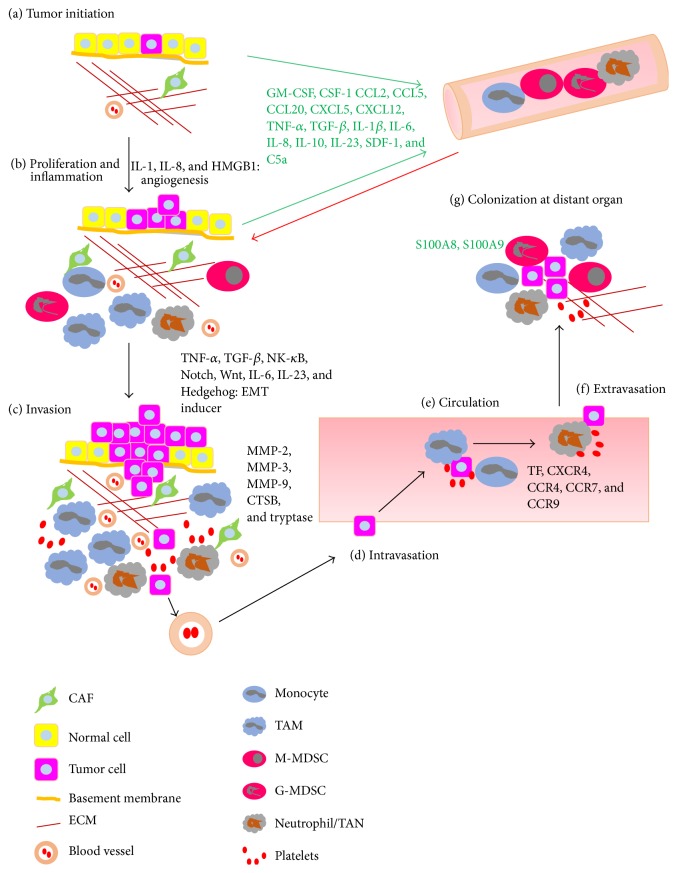
Recruitment pattern of myeloid cells in tumor progression and metastasis. Stages in tumor progression and metastasis including initiation, proliferation and tumor site inflammation, invasion, intravasation, circulation in blood stream, extravasation, and colonization are shown with associated myeloid cells, platelets, and cytokines. The contribution of TAM and TAN at early stage of distant colonization sites is not clear.* Green*: cytokines/chemokines in recruitment or suppression of immune cells,* black*: metastasis associated proteins,* red arrow*: movement of myeloid cells, EMT: epithelial mesenchymal transition, and CTSB: cysteine protease cathepsin B based on [105].

It is now known that chemokines (e.g., CCL2: monocyte chemotactic protein 1), cytokines (e.g., colony-stimulating factor-1 (CSF-1)), and products of the complement cascade are major determinants of macrophage recruitment and positioning in tumors (Figure 2) [40–43]. Simply stated, peripheral blood monocytes are recruited locally and differentiate into macrophages in response to a wide spectrum of chemokines and growth factors produced by stromal and tumor cells in the TME [41]. Do TAMs differentiate only from monocytes recruited from peripheral blood? Lung alveolar and peritoneal macrophages, Kupffer cells, epidermal Langerhans cells, and brain microglia are derived from primitive yolk sac precursors and can be self-maintained locally. These are referred to as tissue-resident macrophages and the evidence that local proliferation of macrophages can contribute to the TAM pool was suggested from a Her2/Neu driven mammary carcinoma animal study [44, 45]. Though we have evidence that both tissue-resident and recruited macrophages may coexist in tumors, that TAMs in a murine mammary tumor model are phenotypically and functionally distinct from mammary tissue-resident macrophages, and also that recruited macrophages may differentiate and form the majority of TAMs, we cannot currently quantify their respective contribution to various stages of progression in many different murine and human tumors [4, 41, 46, 47]. Recently, CSF-1 whose expression was controlled by STAT1 was reported to play an important role at several levels of the monocyte-to-macrophage differentiation pathway in tumors, implying M-CSFR and GM-CSFR signaling in governing the phenotype of macrophage subsets in tumors [45, 48]. Currently, the precise origin of TAMs is thought to be either bone marrow [47] or extramedullary hematopoiesis-like spleen [49] in several studies, indicating that the dominant origin of TAMs appears to be tumor type- or stage-dependent. Overall, the understanding of both of the origin of TAMs and mechanism of their recruitment and differentiation is not completely clear.

## 4. General Characteristics of TANs

In inflamed tissues, neutrophils engage in sophisticated bidirectional interactions with macrophages, dendritic cells, natural killer cells, lymphocytes, and mesenchymal stem cells [50]. However, the interactions have not been significantly understood in the TME. Traditionally, the mechanism of recruitment and function of neutrophils and platelets have been studied mostly in inflammation or bleeding. Neutrophils account for about 60% of all leukocytes in the circulation and are usually the first line of defense at the site of infection or inflammation. Contrary to the well-known ability of inflammatory neutrophils to engulf bacteria, activate the immune system, and induce tissue damage in infections, it appears that TANs can function as immunosuppressive cells in the context of tumors [51]. Neutrophils may influence the phenomenon of macrophage differentiation into pro- or anti-inflammatory subtypes indicated from many studies showing that activated neutrophils, by releasing various chemokines, activate and recruit monocytes/macrophages at the site of inflammation [52]. Besides cytokines, neutrophils also secrete myeloperoxidase (MPO), also important for recruitment of monocytes/macrophages and activation of platelets [53]. These findings and some epidemiological studies indicate that the recruitment and function of neutrophils and platelets may be linked, either directly or indirectly, with those of TAMs and that they are important in cancer progression and also possibly in maintenance of the TME.

Recently, the neutrophil-to-lymphocyte ratio used in combination with elevated platelet count was found to be predictive of the future clinical course of colorectal cancer [54], and, as mentioned, products of the complement cascade are major determinants of macrophage recruitment and positioning in tumors [40–42]. Indeed, TANs have been suggested as key players in malignant transformation, tumor progression, antitumoral immunity, and angiogenesis [50]. It has been suggested that TANs from early tumors are more cytotoxic toward tumor cells and produce higher levels of TNF-*α*, NO, and H_2_O_2_ and, in established tumors, these functions are downregulated and TAN acquire a more protumorigenic phenotype [55]. Neutrophil depletion in two murine models of melanoma and fibrosarcoma reverts the increased tumor growth, angiogenesis, and metastasis observed in IFN-*β*-deficient mice with skewed N2 phenotypes [56], and recent review of the relationship between TAN infiltration and prognosis in human cancer demonstrates the function of TANs in murine and human tumor progression [57]. It is increasingly becoming clear and important that TANs and their myeloid precursors (peripheral neutrophils and granulocytic MDSCs [G-MDSCs]) in the spleen, bone marrow, and blood have important roles in cancer biology [58]. Neutrophils also make up a significant portion of the inflammatory cell infiltrate in many models of cancer, though they release far less cytokine when compared with other myeloid cells in the TME [59]. It was reported that, at early stages of tumor development, neutrophils are almost exclusively at the periphery of the tumor [55]. At later stages, neutrophils are also found scattered among the tumor cells.

Studies have shown that, analogously to the M1 and M2 dichotomy, TANs develop a protumorigenic (N2) phenotype in untreated tumors, largely driven by the presence of TGF-*β* [58], and that blocking the effects of TGF-*β* or augmenting IFN-*β* can also alter the phenotype of TANs to a more antitumor (N1) phenotype [56]. Antitumor “N1-like” cells generated in the absence of TGF-*β* produced higher levels of TNF-*α*, MIP-1*α*, H_2_O_2_, and NO and were cytotoxic to tumor cells both* in vitro* and* in vivo* [59].

Respiratory burst and granule proteins are two main mechanisms of cell killing by neutrophils. Transcriptome analysis of naive bone marrow neutrophils (NN) from nontumor bearing mice and G-MDSC and TAN from mice in which AB12 mesothelioma tumors were growing showed that expression levels of both proteins involved in respiratory burst and granule proteins were downregulated and that those of chemokine, cytokine, and APC genes were upregulated in TANs [58]. N2-like neutrophils may also synergistically interact with tumor-resident mesenchymal stem cells (MSCs) to prompt cancer progression [60]. TANs from established tumors produce CCL17 or CCL22, recruiting immunosuppressive regulatory T cells (Tregs) with defective cytotoxic functions into the tumor and leading to suppression of antitumoral immunity [61]. Of note, similarly to TAMs, TANs from early tumors were more cytotoxic toward tumor cells, while in established tumors TANs acquire a more protumoral phenotype, showing how the evolvement of the TME influences TAN phenotype [55]. Unlike TAMs, it is not certain whether activation of TANs is reversible, and it has been suggested that N1-like and N2-like phenotypes of neutrophils may be from different degrees of activation rather than polarization [62]. The important question whether TANs can be manipulated to undergo frank irreversible activation or possibly reversible activation states remains unresolved and should be a matter of further research.

## 5. Recruitment of TANs

Do we know the origin of TANs? It is known that the spleen is the site of localization of TAM and TAN precursors, from where they physically relocate to the tumor stroma, and that CXCL8 (IL-8), a chemoattractant for neutrophils, is also chiefly responsible for the recruitment of TANs (Figure 2) [49]. A recent transcriptome study showed that TANs are not “tissue-based G-MDSCs” modulated by the TME but are a different population of neutrophils from both bone marrow-derived neutrophils and G-MDSCs [58]. However, we are not sure whether the majority of TANs are actually differentiated from G-MDSCs that have been recruited to the tumor or whether they are bone marrow-/blood-derived neutrophils, converted to N2 TANs in the TME specifically by the high local concentrations of TGF-*β* [63]. Though the study does not clarify whether the cells were recruited from the bone marrow/blood pool of neutrophils or the splenic G-MDSC population, the two studies support the idea that TGF-*β* and other factors in the TME may affect the local “education” of recruited neutrophils.

## 6. Possible Interaction of TANs with TAMs

Do TANs then recruit TAM precursors to the tumor site or are they responsible for the M2-like activation of macrophages in the TME? It is known that activated neutrophils releasing IL-8 and TNF-*α* activate and recruit macrophages at the site of inflammation [64]. Neutrophils secrete MPO, and MPO binding to the MMR induces secretion of reactive oxygen intermediates, IL-8, TNF-*α*, and GM-CSF in chronic inflammatory environments such as rheumatoid joints [65]. M2-like macrophages express high levels of macrophage mannose receptor (MMR) and IL-10 and low levels of HLA-DR and IL-1*β* [66]. Though we still lack direct evidence that supports TAN and TAM interaction through MPO and the MMR, massive MPO-positive neutrophil infiltration has been found in established colorectal cancer [67] and lung cancer [68]. Also, similar influence of TGF-*β* on activation of macrophages and neutrophils (M2-like and N2-like, resp.) indicates a close link between TAMs and TANs in the same TME and the possibility that recruitment of macrophages by neutrophils may precede their N2-like polarization. It would be necessary to confirm whether the interaction between TANs and TAMs in the TME is similar to well-known interactions between neutrophils and macrophages in a nontumoral chronic inflammatory environment.

## 7. Nuclear Extracellular Trap (NET) Formation in the TME

NETs are neutrophil-derived structures composed of decompacted chromatin (DNA and citrullinated associating histones) and antimicrobial peptides, and NET-producing “NETosis” is a form of neutrophil death, distinct from apoptosis or necrosis [69]. NETs are introduced to trap and kill microorganisms and facilitate a final form of neutrophil-mediated host defense against microorganisms. They have also been found in non-microorganism-induced inflammatory environments in autoimmune diseases such as systemic lupus erythematous (SLE) and rheumatoid arthritis (RA) [70–73] and tumors [74, 75]. In autoimmune diseases such as RA, neutrophils are mostly responsible for the cytotoxic effects of immune cells and NETs appear to provide autoantigens and mediate organ damage [70, 76]. However, the function of NETs in tumor progression is still not clear, although they have been suggested to contribute to metastasis from trapping of circulating tumor cells at distant metastatic sites [74, 77] and to tumor progression at primary sites by providing a high local concentration of biologically active proteins [75, 77]. The available data indicate a lack of evidence to conclusively demonstrate whether TANs actually produce NETs and to indicate which signaling is involved in NETosis in the TME. Though we know the relationship between deposition of NETs and recruitment of MPO-rich population of neutrophils in tumors, it seems that there is not enough evidence to indicate the existence of TAN specific NETosis [74, 75, 78]. The animal studies were performed with infusion of bone marrow- or spleen-derived naive neutrophils and the localization of general neutrophils, not specifically TANs, was characterized from MPO staining in tumor. The recent identification of TAN specific signatures such as CD62L^lo^CD54^hi^ phenotype with a distinct repertoire of chemokine receptors including CCR5, CCR7, CXCR3, and CXCR4 in human lung cancer indicates that further study to validate TAN specific NETosis may be possible in animal studies [79]. Another function of NETs is to provide autoantigens. In SLE and RA patients, specific autoantigens, such as anti-dsDNA and anti-citrullinated protein antibodies and rheumatoid factor, respectively, have been detected. However, there seems to be a relative paucity of tumor-derived autoantigens identified thus far, and this suggests that a major function of tumoral NETs is more likely to trap migrating tumor cells and to provide protumoral substances rather than immunomodulating autoantigens. Still, it is becoming clear that NETs are a very recently introduced component of the TME and that they play another protumoral role in tumor progression. Future studies will probably investigate (i) identification of the N1-like, N2-like, or general neutrophils that actually form NETs and the specific tumor progression stage to which NETs primarily contribute; (ii) whether retention of TAMs or TANs in tumors also requires formation of NETs; (iii) whether M2-like or N2-like activation requires NETs; (iv) which signals are involved in the formation of NETs.

## 8. Platelets as a Potential Hub for the Recruitment of Macrophages and Neutrophils

Platelets also contribute to tumor progression [80, 81]. High platelet count in blood (thrombocytosis) is associated with decreased survival in a wide range of cancers including breast, colorectal, and lung cancer [82, 83]. An increased platelet count in blood in malignancy is associated with poor patient prognosis [84, 85]. It has been suggested that platelets may protect tumor cells from immune attack in the circulation, may provide adhesive sites for tumor dissemination, may provide chemokine signals for macrophage recruitment in tumors, and may even shuttle growth factors and cytokines from one site to another [2]. By forming microthrombi, platelets may function as a “shield” to protect disseminating cancer cells in microcirculation from immune cell attack. Platelets store various chemokines and the majority (~80%) of VEGF detectable in blood and platelets induces angiogenesis* in vivo* [85].

**Figure 3:**
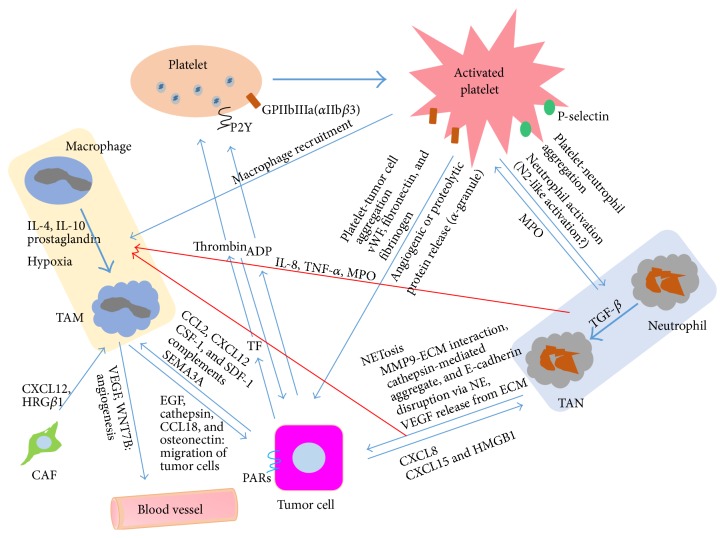
Summary of representative interactions between TAM, TAN, platelet, and tumor cells. The interactions between neutrophil and macrophages have not been significantly understood in the TME and the contribution of platelet in differentiation of TAM and TAN suggested in this review awaits further studies. Tumor cells, blood vessels, and CAF comprise TME. CCL2, CXCL12, CSF-1, SDF-1, complements, and SEMA3A for macrophage recruitment [30, 106]. CSF-1 prompts TAMs to produce EGF. The EGF-CSF-1 loop can be initiated by CAF derived factors, such as CXCL12 and HRG*β*1 [106]. IL-4 from CD4+ T cells or tumor cells can activate macrophages to TAMs. CCL18 and osteonectin can increase migration and intravasation of tumor cells in metastasis. CXCL-8, CXCL15, and HMGB1 secreted from tumor cells can recruit TANs in metastatic sites. MPO and cytokines from neutrophil recruit platelet and macrophages. PAR and P2Y receptor are involved in thrombin and ADP mediated platelet activation, respectively. P-selectin is involved in platelet leukocyte tethering and leukocyte activation. *α*-granule is a storage of proteins that enhance adhesive process, angiogenesis, and extracellular matrix (ECM) degradation [81]. GPIIbIIIa mediates tumor cell and platelet interaction via vWF, fibronectin, and fibrinogen [80].* Red arrow*: neutrophil-mediated recruitment of macrophages in tumor.* Thick arrow*: conversion of platelets, neutrophils, and macrophages to activated platelets, TAN, and TAM, respectively. GPIIbIIIa, glycoprotein IIbIIIa; vWF, Von Willebrand factor; ADP, adenosine diphosphate; PARs, proteinase-activated receptors; P2Y, P2Y receptors; TF, tissue factor; NE, neutrophil elastase; HMGB1, high mobility group protein B1; HRG*β*1, heregulin *β*1.

Platelets play key roles in directing homing and retention signals for bone marrow-derived cells (BMDCs) and cancer cells and also secrete SDF-1, critical for macrophage recruitment and positioning in tumors [2]. Also, platelet-derived SDF-1 is critical for migration of CXCR4+ tumor cells, hematopoietic progenitor cells (HPCs), and endothelial progenitor cells (EPCs) [86]. This is meaningful in that BMDCs homing to the primary tumor niche may remain in an undifferentiated state in the form of HPCs, EPCs, MSCs, or GR-1+CD11b+ MDSCs or may differentiate into more specialized cell types including TAMs [2]. It is known that platelets support the recruitment of leukocytes in inflammation and vice versa and that the interaction between platelets and neutrophils can happen not only at the inflammatory site, but also in the circulation, indicating the role of platelets in metastasis [87]. Platelets can recruit themselves and neutrophils via various mechanisms, such as the formation of platelet/leukocyte complexes, secretion of serotonin, and induction of P-selectin on platelets and ICAM-1 and *α*v*β*3 on endothelial cells [87]. All of these findings indicate that platelets may play a central role in recruiting neutrophils in a chronically “persistent” inflammatory environment, that is, the TME. Tumor cells express tissue factor (TF), which is a receptor for coagulation factors VIIa and X [88, 89]. Clot formation by TF expressed by tumor cells enhances recruitment of macrophages in a lung metastasis model through various mechanisms including protease-activated receptor [90], and recruitment of granulocytic cells by the platelet-secreted CXCR2 ligands, CXCL5 and CXCL7 chemokines, upon platelet contact with tumor cells is essential mechanism for the guidance of granulocytes to form “early metastatic niches” [81, 91, 92]. Importantly, recent results indicate that complement components and platelets are key players in cancer-related inflammation and mediate recruitment of macrophages at least partially via CCL2 [40]. Summary of representative interactions between TAM, TAN, and platelets described in this review can be found in Figure 3.

All of this evidence emphasizes the role of platelets in recruitment of macrophages and neutrophils in tumor sites. Though we still lack evidence to support the role of platelets in activation of macrophages and neutrophils—and it is generally accepted that their tumor-protective role in the blood stream may be the most profound influence of platelets on tumor progression—thrombocytopenic mice show increased blood TNF-*α* and IL-6 and decreased TGF-*β* [87], possibly favoring antitumoral polarization; as stated, platelets are involved in recruitment of both macrophages and neutrophils in both primary and metastatic tumor sites. At present, there are important questions to be solved: which stages in tumor progression, including metastasis, are primarily affected by platelet functions, which of the adhesive or paracrine functions of platelets are more important for tumor progression, and which platelet factor or traditionally emphasized tissue factor is more important for the protumoral activity of the coagulation system? Further research will likely demonstrate the functional contribution of platelets in tumor progression, including the development of protumoral TAMs and TANs.

## 9. Clinical Implications

All the summarized data describing the protumoral role of the myeloid infiltrate of tumors in this review emphasize that TAMCs are reasonable targets for new anticancer therapeutic approaches. It is now becoming clear that host-protective properties of macrophages are suppressed in the TME and that therapeutic intervention can reverse this suppression. Recently explored strategies have focused on ablation of macrophages or reduction of recruitment of myeloid cells and repolarization of M2-like protumoral macrophages to antitumoral M1-like cells. CD40 agonist antibody [93] and TLR9 agonist (CpG-oligodeoxynucleotide) [94] have been shown to be effective in repolarizing M2-like protumoral macrophages. CCL2/CCR2 antagonist [95, 96] and CSF-1 inhibitory antibodies or Yondelis (trabectedin) [97] were effective in blocking recruitment of macrophages in tumor sites. Bisphosphonate zoledronic acid [36, 98] and clodronate [99] have been used to inhibit TAM effectors and to deplete TAMs, respectively.

Rather than depleting the entire population of neutrophils, the usual strategy is to deplete TANs or disrupt their homing ability, migration. For this purpose, the deployment of anti-CXCR2 antibodies to deplete TANs or the targeting of specific neutrophil-derived or recruiting chemokines, such as CXCL-5, Gro-*α*, or IL-8, was performed and reported to be successful [59]. Furthermore, targeting TGF-*β* or augmenting the activity of IFN-*β* to block its skewing of TANs toward an N2 phenotype may have potential as a new therapeutic approach [56, 63]. As neutrophil-derived molecules play critical roles in a wide range of stages of tumor progression [59], targeting neutrophil-secreted enzymes or cytokines could be another effective approach [100]. Targeting TANs may indirectly affect TAM populations, considering the interaction between neutrophils and macrophages mentioned above.

Because aggressive anticoagulant therapy in cancer patients carries the risk of bleeding complications, selective inhibition of TF signaling or platelet functions should be considered for clinical settings. Currently, the benefit that direct platelet receptor antagonists may have on cancer prognosis has not been demonstrated, and the evidence to support a combined use of antiplatelet agents with current chemotherapeutic reagents is lacking [101]. The concept that tumor cells alter their gene expression profiles to acquire a genophenotype closely resembling that of platelets and express several megakaryocytic genes (adhesion receptors *α*IIb*β*3, thrombin receptor, and PECAM/CD31 and/or platelet-type 12-LOX) to activate platelets or the coagulation cascade is referred to as “platelet mimicry” of tumor cells [102]. This well-described epiphenomenon facilitates hematogenous dissemination of tumor cells in metastasis; thus, identification of molecular targets to regulate platelet mimicry is also likely to provide new therapeutic modalities. Recently, the CXCR2 receptor for the granulocyte- and platelet-derived ligand CXCL5/7 was shown to be important for recruitment of neutrophils to early metastatic niches [92], and CXCR2 inhibitors reduce the recruitment of granulocytes in primary tumor sites as well [103, 104]. Considering that anti-CXCR2 inhibitors evaluated in the clinic for inflammatory disease are well tolerated by most patients [103], targeting the CXCR2-CXCL5/7 axis may become effective in clinical settings.

## 10. Conclusions and Perspectives

There has been tremendous effort and progress in deciphering the function of myeloid cells in the TME and in tumor progression by excellent investigators in the field. Unfortunately, the wealth of mounting information regarding protumoral myeloid cells in cancer has been fragmentary and produced confusion from a disjointed view of the role of macrophages and neutrophils in the TME [19]. In addition, the absence of unique markers to differentiate each subset has obscured the nature of specific myeloid subsets in cancer. However, the contribution of TAMs and TANs to tumor progression is clear, and animal model-based or preclinical studies have shown promising results. We anticipate that the introduction of more sophisticated tumor models and techniques to differentiate different myeloid cell subsets* in vivo* will reveal fundamental information about possible spatial and temporal modulation of myeloid cells, including their interaction with platelets in each progression stage of different types of tumors.
